# Supplementary material for: Taxonomic Review of the Genus Caloptilia Hübner, 1825 (Lepidoptera: Gracillariidae) with Descriptions of Three New Species and Seven Newly Recorded Species from Korea
Source: Insects. 2022 Nov 30;13(12):1107. doi: 10.3390/insects13121107 (PMC9785696; doi:10.3390/insects13121107)
Supplement: Supplementary file 1 [file insects-13-01107-s001.zip › File S1 Annotated checklist.pdf]

Supplementary Material 2. Annotated checklist for the Korean *Caloptilia* species

Order **LEPIDOPTERA** Linnaeus, 1758
Superfamily **GRACILLARIOIDEA** Stainton, 1854
Family **GRACILLARIIDAE** Stainton, 1854
Gracillariidae Stainton, 1854. Insecta Britannica, Lepidoptera, Tineina: 193.
Type genus: *Gracillaria* Haworth, 1828.

Genus ***Caloptilia*** Hübner, 1825
Caloptilia Hübner, 1825: 427.
Type species: *Tinea upupaepennella* Hübner, 1796.
Antiolopha Meyrick, 1864.
Calliptilia Agassiz, 1847.
Cecidoptilia Kumata, 1982.
Coriscium Zeller, 1839.
Minyoptilia Kumata, 1982.
Ornix Treitschke, 1833.
Ornix Kollar, 1832.
Phylloptilia Kumata, 1982.
Poeciloptilia Hübner, 1825.
Rhadinoptilia Kumata, 1982.
Sphyrophora Vári, 1961.
Timodora Meyrick, 1886.

1. *Caloptilia acericola* Kumata, 1966 긴노랑가는나방

Caloptilia acericola Kumata, 1966: 2-3. TL: Hokkaidō, Japan. TD: EIHU (Holotype; Paratypes).

Distribution. Korea (new record), Japan, Russia.

Host plants. *Acer japonicum* Thunb., *A. palmatum* Thunb., *A. pictum* Thunb. ex Murray, *A. pseudosieboldianum* (Pax) Kom. [Sapindaceae].

2. *Caloptilia aceris* Kumata, 1966 단풍잎가는나방

Caloptilia aceris Kumata, 1966: 1. TL: Hokkaido, Japan. TD: EIHU (Holotype; Paratypes).

Distribution. Korea, China, Japan, Russia.

Host plants. *Acer miyabei* Maxim., *A. palmatum* Thunb., *A. pictum* Thunb. ex Murray, *A. saccharum* Marschall [Sapindaceae]

3. *Caloptilia alni* Kumata, 1966 오리나무가는나방

Caloptilia alni Kumata, 1966: 12. TL: Hokkaidō, Japan. TD: EIHU (Holotype; Paratypes).

Distribution. Korea, China, Japan, Russia.

Host plants. *Alnus hirsuta* Turcz., *A. japonica* (Thunb.) Steud. [Betulaceae].

4. *Caloptilia azaleella* (Brants, 1966) 산철쭉가는나방

Gracillaria azaleella Brants, 1966: lxx–lxii. TL: Japan. TD: Unknown.

Gracillaria anthracosperma Meyrick, 1931.

Gracillaria azaleae Busck, 1914.

Distribution. Korea, China, Japan, Russia, Australia, New Zealand, Austria, Belgium, Czech Republic, Denmark, Finland, France, Germany, Hungary, Ireland, Italy, Luxembourg, Netherlands, Norway, Poland, Portugal, Slovakia, Sweden, Switzerland, United Kingdom, Canada, United States, South Africa.

Host plants. *Rhododendron yedoense* var. *poukhanense*, *R. decandrum* Makino, *R. indicum* (L.) Sweet, *R. kaempferi* Planch, *R. kiusianum* Makino, *R. macrosepalum* Maxim. *R. viscistylum*, *R.* simsii Planch., *R.* japonicum Sur., *R.* obtusum (Lindl.) Planch, *R*. sp., *Azalea* sp. [Ericaceae].

5. *Caloptilia celtidis* Kumata, 1982 흰무늬가는나방

Caloptilia celtidis Kumata, 1982: 76-79. TL: Honshū, Japan. TD: EIHU (Holotype); EIHU, BMNH (Paratypes).

Distribution. Korea (new record), China, Hong Kong, Japan.

Host plants. *Celtis sinensis* Persoon, *C. jessoensis* Koidz. [Cannabaceae].

Remarks. This species reared from *Celtis sinensis* Persoon of the family Cannabaceaee in this study.

6. *Caloptilia chrysolampra* (Meyrick, 1938) 버들가는나방

Gracillaria chrysolampra Meyrick, 1936: 38. TL: Taiwan. TD: BMNH (Syntypes).

Distribution. Korea, China, Japan, Taiwan.

Host plants. *Salix pseudo-lasiopyne*, *S. babylonica* L., 1753, *S.* sp., *Populus nigra* L., *P. nigra* L. [Salicaceae].

7. *Caloptilia dentata* Liu & Yuan, 1990 네모무늬가는나방

Caloptilia (Caloptilia) dentata Liu & Yuan, 1990: 186. TL: Beijing, China. TD: IZAS (Holotype; Paratypes).

Distribution. Korea (new record), China.

Host plants. *Acer truncatum* Bunge [Sapindaceae].

8. *Caloptilia hidakensis* Kumata, 1966 고로쇠가는나방

Caloptilia hidakensis Kumata, 1966: 4. TL: Hokkaidō, Japan. TD: EIHU (Holotype; Paratype).

Distribution. Korea, Japan, Russia.

Host plants. *Acer pictum* Thunb. ex Murray [Sapindaceae]

9. *Caloptilia kadsurae* Kumata, 1966 남오미자가는나방

Caloptilia kadsurae Kumata, 1966: 19. TL: Honshū, Japan. TD: EIHU (Holotype; Paratypes).

Distribution. Korea (new record), Japan.

Host plants. *Kadsura japonica* Dunal [Magnoliaceae].

Remarks. This species reared from *Kadsura japonica* Dunal of the family Magnoliaceae in this study.

10. *Caloptilia kisoensis* Kumata, 1982 신나무가는나방

Caloptilia (Caloptilia) kisoensis Kumata, 1982: 45-47. TL: Honshū, Japan. TD: EIHU (Holotype; Paratypes).

Distribution. Korea, Japan, Russia.

Host plants. *Acer ginnala* Maxim., *A. pictum* Thunb. ex Murray [Sapindaceae].

11. *Caloptilia koreana* Kim and Byun, 2022 한국가는나방

Caloptilia koreana Kim and Byun, 2022. TL: Gwangneung, Korea. TD: HNUSEL (Holotype).

Distribution. Korea (endemic).

Host plants. Unknown.

12. *Caloptilia leucothoes* Kumata, 1982 산진달래가는나방

Caloptilia (Caloptilia) leucothoes Kumata, 1982: 68 [2]. TL: Hokkaido, Japan. TD: EIHU (Holotype), EIHU, BMNH (Paratypes).

Distribution. Korea, Japan, Russia.

Host plants. *Leucothoe grayana* Maxim., *Rhododendron albrechti* Maxim., *R. dauricum* L., *R. dilatatum* Miq., *R. reticulatum* D. Don, *R.* sp., *Menziesia pentandra* Maxim. [Ericaceae].

13. *Caloptilia magnoliae* Kumata, 1966 목련가는나방

Caloptilia magnoliae Kumata, 1966: 17 [17]. TL: Hokkaido, Japan. TD: EIHU (Holotype; Paratypes).

Distribution. Korea, Japan.

Host plants. *Machilus thunbergii* Siebold & Zucc. [Lauraceae], *Magnolia* sp., *M. kobus* DC [Magnoliaceae].

14. *Caloptilia mandschurica* (Christoph, 1882) 북방민가는나방

Gracillaria mandschurica Christoph, 1882: 39-40. TL: Russia. TD: BMNH (Holotype; Allotype).

Caloptilia (Caloptilia) mongolicae: Kumata, 1982.

Distribution. Korea, China, Japan, Russia.

Host plants. *Quercus mongolica* subsp. *crispula* (Blume), *Castanea crenata* Siebold & Zucc., *Q. acutissima* Carruth., *Q. dentata* Thunb., *Q. crispula* Blume, *Q. serrata* Thunb. [Fagaceae].

Remarks. This species reared from *Quercus mongolica* subsp. *crispula* (Blume) of the family Fagaceae in this study.

15. *Caloptilia monticola* Kumata, 1966 외반점가는나방

Caloptilia monticola Kumata, 1966: 8. TL: Honshu, Japan. TD: EIHU (Holotype; Paratypes).

Distribution. Korea (new record), China, Japan, Russia.

Host plants. *Acer argutum* Maxim., *A. ginnala* Maxim., *A. pentaphyllum* Diels., *A. pictum* Thunb. ex Murray, *A. rufinerve* Siebold & Zucc., *A. semenovii* Regel & Herd, *A. ukurunduense* Trautv. & Mey., *A. distylum* Siebold & Zucc., *A. micranthum* Siebold & Zucc., *A. tschonoskii* Maxim., *A. ukurunduense* Trautv. & Mey., *A. semenovii* Regel & Herd, *A.* sp. [Sapindaceae].

16. *Caloptilia pulverea* Kumata, 1966 흑갈색점가는나방

Caloptilia pulverea Kumata, 1966:13 [17]. TL: Hokkaido, Japan. TD: EIHU (Holotype), EIHU, BMNH (Paratypes).

Distribution. Korea, China, Japan, Russia.

Host plants. *Alnus hirsuta* Turcz., *A. japonica* (Thunb.) Steud., *A. matsumurae* Callier, *A. maximowiczii* Callier, *A. rugosa* (Du Roi) Spreng. *A. firma* Siebold & Zucc., *A. serratuloides* Callier [Betulaceae].

17. *Caloptilia purpureus* Kim and Byun, 2022 보라가는나방

Caloptilia purpureus Kim and Byun, 2022. TL: Yeosu, Korea. TD: HNUSEL (Holotype).

Distribution. Korea (endemic).

Host plants. *Sageretia theezans* (L.) Brongn. [Rhamnaceae].

Remarks. This species reared from *Sageretia theezans* (L.) Brongn. of the family Rhamnaceae in this study.

18. *Caloptilia pyrrhaspis* (Meyrick, 1931) 반무늬가는나방

Gracillaria pyrrhaspis Meyrick, 1931: 17. TL: Szechuan, China. TD: BMNH (Holotype).

Caloptilia bicolor Ermolaev, 1977: 105, 110.

Caloptilia pyrrhaspis: Kumata, 1982: 74.

Distribution. Korea, China, Japan, Russia.

Host plants. *Betula davurica* Pall., *B. ermanii* Chamisso, *B. grossa* Siebold & Zucc., *B. dahurica* Pall., *B. platyphylla* Sukaczev [Betulaceae].

Remarks. This species reared from *Betula davurica* Pall. of the family Betulaceae in this study.

19. *Caloptilia recitata* (Meyrick, 1918) 큰갈색무늬가는나방

Gracillaria recitata Meyrick, 1918b: 178-179. TL: Assam, India. TD: BMNH (Syntypes).

Caloptilia recitata: Issiki, 1957: 30.

Distribution. Korea (new record), China, Hong Kong, Japan, India, Nepal.

Host plants. *Cotinus coggygria* Scop., *Rhus javanica* L., *Toxicodendron sylvestre* (Siebold & Zucc.) Kuntze, *T. trichocarpum* (Miq.) Kuntze in China [Anacardiaceae].

Remarks. This species collected from *Ailanthus altissima* (Mill.) [Simaroubaceae] with pupal cocoon on the back side.

20. *Caloptilia rhois* Kumata, 1982 옻나무가는나방

Caloptilia (Caloptilia) rhois Kumata, 1982: 62-65. TL: Honshu, Japan. TD: EIHU (Holotype; Paratypes).

Distribution. Korea, China, Japan, Hong Kong.

Host plants. *Rhus javanica* L., *Toxicodendron succedaneum* (L.) Kuntze [Anacardiaceae].

21. *Caloptilia sapporella* (Matsumura, 1931) 졸참나무가는나방

Gracillaria sapporella Matsumura, 1931: 1101. TL: Japan. TD: EIHU (Syntype).

Caloptilia illicii Kumata, 1966: Shin et al., 2015 (misidentification).

Distribution. Korea, China, Japan, Russia.

Host plants. *Quercus acutissima* Carruth., *Castanea crenata* Siebold & Zucc., *Q. dentata* Thunb., *Q. mongolica* subsp. *crispula* (Blume) Menitsky, *Q. serrata* Thunb., *Q. crispula* Blume [Fagaceae].

22. *Caloptilia schisandrae* Kumata, 1966 오미자가는나방

Caloptilia schisandrae Kumata, 1966: 18. TL: Hokkaido, Japan. TD: EIHU (Holotype; Paratypes).

Distribution. Korea, China, Japan, Russia.

Host plants. *Schisandra chinensis* Baill. [Magnoliaceae].

23. ***Caloptilia soyella*** **(van Deventer, 1904) 톱니가는나방**

Gracillaria soyella van Deventer, 1904: 22-25. TL: Java, Indonesia. TD: RNHL (Lectotype; Paralectotype).

Caloptilia soyella: Issiki, 1950: 451.

Distribution. Korea (new record), China, Japan, India, Indonesia, Viet Nam, Fiji, Cape Verde.

Host plants. *Lespedeza cyrtobotrya* Miq. [Fabaceae], *Cajanus cajan* (L.) Millsp., *Glycine max* (L.) Merr., *Kummerovia striana* Schindler, *Phaseolus mungo* L., *Vigna angularis* (Willd.) Ohwi & H. Ohashi, *P. calcaratus* Roxb., *Soya hispida* Moench [Fabaceae].

Remarks. This species reared from *Lespedeza cyrtobotrya* Miq. of the family Fabaceae in this study.

24. ***Caloptilia stigmatella*** **(Fabricius, 1781) 백양나무가는나방**

Tinea stigmatella Fabricius, 1781: 295-296 . TL: United Kingdom. TD: GLAHM (Holotype).

Gracillaria consimilella Frey & Boll, 1876.

Phalaena cruciella Goeze, 1783.

Tinea equestris de Fourcroy, 1785.

Gracillaria ochracea Haworth, 1828.

Gracillaria purpurea Haworth, 1828.

Gracillaria purpuriella Chambers, 1872.

Tinea triangulella Panzer, 1794.

Tinea triangolosella Costa, 1836.

Gracillaria trigona Haworth, 1828.

Tinea upupaepennella Hübner, 1791.

Distribution. Korea, China, Japan, Russia, Mongolia, India, Armenia, Austria, Belgium, Bosnia and Herzegovina, Bulgaria, Croatia, Czech Republic, Estonia, Finland, France, Georgia, Germany, Hungary, Ireland, Italy, Kyrgyzstan, Latvia, Leichtenstein, Lithuania, Luxembourg, Macedonia, Morocco, Netherlands, Norway, Poland, Portugal, Romania, Serbia, Slovakia, Spain, Sweden, Switzerland, Tajikstan, Turkey, Turkmenistan, Ukraine, Uzbekistan, United Kingdom, Canada, United states.

Host plants. *Populus* sp., *P. nigra, P. alba* L., *P.* sp., *Salix bakko* Kimura, *Salix miyabeana* (Seemen), *S.* sp., L., *S. lanata* L., *S. pedicellata* Desf., *S. triandra* L., *S. fragilis* L., *S. purpurea* L., *S. atrocinerea* Brot., *S. salviifolia* Brot., *S. longifolia* Lam., *S. repens* L., *S. caprea* L., *S. sachalinensis* Schmidt & Sekka, *S. alba* L., *S. eleagnos* Scop., *S. cinerea* L., *S. incanaelaeagnos* Scop [Salicaceae], *Robinia pseudacacia* L. [Fabaceae], *Myrica gale* L., [Myricaceae].

25. ***Caloptilia syrphetias*** **(Meyrick, 1907) 후박나무가는나방**

Gracilaria syrphetias Meyrick, 1907: 984. TL: Ceylon, Sri Lanka. TD: NHMUK.

Gracilaria zopherotarsa: Meyrick, 1936: 39.

Caloptilia perseella: Kumata, 1982: 93.

Distribution. Korea (Jeju Island), Brunei Darussalam, China, Hong Kong, India, Indonesia, Japan, Malaysia, Sri Lanka, Thailand.

Host plants. *Machilus thunbergii* Siebold et Zucc. [Lauraceae].

26. *Caloptilia theivora* (Walsingham, 1891) 동백가는나방

Gracillaria theivora Walsingham, 1891: 49-50. TL: Ceylon, Sri Lanka. TD: BMNH (Lectotype; Paralectotype).

Caloptilia theivora: Issiki, 1950

Distribution. Korea, China, Hong Kong, Japan, Brunei Darussalam, India, Indonesia, Malaysia, Sri Lanka, Taiwan, Thailand, Viet Nam.

Host plants. *Camellia sinensis* L., *C. japonica* L., *C. sasanqua* Thunb., *Thea sinensis* L., *C. theifera* Griff., [Theaceae].

Remarks. This species reared from *Camellia sinensis* L. of the family Theaceae in this study.

27. *Caloptilia xanthos* Kim and Byun, 2022 노랑점가는나방

Caloptilia xanthos Kim and Byun, 2022. TL: Muan-gun, Korea. TD: HNUSEL (Holotype).

Distribution. Korea (endemic).

Host plants. Unknown.

28. *Caloptilia yasudai* Kumata, 1982 노란이빨가는나방

Caloptilia yasudai Kumata, 1982: 51-53. TL: Hokkaido, Japan. TD: EIHU (Holotype; Paratyeps).

Distribution. Korea, Japan.

Host plants. Unknown.

Remarks. This species cannot be examined in this study.

29. *Caloptilia zachrysa* (Meyrick, 1907) 사과잎가는나방

Gracillaria zachrysa Meyrick, 1907: 983. TL: Ceylon, Sri Lanka. TD: BMNH (Syntypes).

Caloptilia zachrysa: Issiki, 1957.

Distribution. Korea, China, Japan, India, Sri Lanka, Taiwan.

Host plants. *Malus pumila* Mill., *M. pumila* Mill., *M. sylvestris* Mill., *Prunus persica* (L.) Batsch, *Photinia*

glabra (Thunb.) Maxim., *Ph*. sp., *Rubus* sp. [Rosaceae].
