# Supplementary material for: Taxonomic Review of the Genus Caloptilia Hübner, 1825 (Lepidoptera: Gracillariidae) with Descriptions of Three New Species and Seven Newly Recorded Species from Korea
Source: Insects. 2022 Nov 30;13(12):1107. doi: 10.3390/insects13121107 (PMC9785696; doi:10.3390/insects13121107)
Supplement: Supplementary file 1 [file insects-13-01107-s001.zip › Table S1 Host plants of the Korean Caloptilia species.pdf]

Supplementary Material Table S1. Host plants of the Korean *Caloptilia* species.

Subfamily	Species	Host plants
Gracillariinae	*Caloptilia acericola*	Sapindaceae
Gracillariinae	*Caloptilia aceris*	Sapindaceae
Gracillariinae	*Caloptilia alni*	Betulaceae
Gracillariinae	*Caloptilia azaleella*	Ericaceae
Gracillariinae	*Caloptilia celtidis**	Cannabaceae†
Gracillariinae	*Caloptilia chrysolampra*	Salicaceae
Gracillariinae	*Caloptilia dentata*	Sapindaceae
Gracillariinae	*Caloptilia hidakensis*	Sapindaceae
Gracillariinae	*Caloptilia kadsurae**	Magnoliaceae†
Gracillariinae	*Caloptilia kisoensis*	Sapindaceae
Gracillariinae	*Caloptilia leucothoes*	Ericaceae
Gracillariinae	*Caloptilia magnoliae**	Lauraceae†, Magnoliaceae
Gracillariinae	*Caloptilia mandschurica**	Fagaceae†
Gracillariinae	*Caloptilia monticola*	Sapindaceae
Gracillariinae	*Caloptilia pulverea*	Betulaceae
Gracillariinae	*Caloptilia pyrrhaspis**	Betulaceae†
Gracillariinae	*Caloptilia recitata*	Anacardiaceae
Gracillariinae	*Caloptilia rhois*	Anacardiaceae
Gracillariinae	*Caloptilia sapporella*	Fagaceae
Gracillariinae	*Caloptilia schisandrae*	Magnoliaceae
Gracillariinae	*Caloptilia soyella**	Fabaceae†
Gracillariinae	*Caloptilia stigmatella*	Salicaceae
Gracillariinae	*Caloptilia theivora**	Theaceae†
Gracillariinae	*Caloptilia yasudai*	Unknown
Gracillariinae	*Caloptilia zachrysa*	Rosaceae
Gracillariinae	*Caloptilia koreana*	Unknown
Gracillariinae	*Caloptilia purpureus**	Rhamnaceae†
Gracillariinae	*Caloptilia xanthos*	Unknown

(*: rearing from host plants, †: new record of host plants)
